# The Relationship between Cognitive Reserve and Math Abilities

**DOI:** 10.3389/fnagi.2017.00429

**Published:** 2017-12-22

**Authors:** Giorgio Arcara, Sara Mondini, Alice Bisso, Katie Palmer, Francesca Meneghello, Carlo Semenza

**Affiliations:** ^1^San Camillo Hospital IRCCS, Venice, Italy; ^2^Department of General Psychology, University of Padua, Padua, Italy; ^3^Human Inspired Technology Research-Centre, University of Padua, Padua, Italy; ^4^Department of Neurosciences (Padova Neuroscience Center), University of Padua, Padua, Italy

**Keywords:** aging, cognitive reserve, maths, mathematical abilities, daily living activities, healthy aging

## Abstract

Cognitive Reserve is the capital of knowledge and experiences that an individual acquires over their life-span. Cognitive Reserve is strictly related to Brain Reserve, which is the ability of the brain to cope with damage. These two concepts could explain many phenomena such as the modality of onset in dementia or the different degree of impairment in cognitive abilities in aging. The aim of this study is to verify the effect of Cognitive Reserve, as measured by a questionnaire, on a variety of numerical abilities (number comprehension, reading and writing numbers, rules and principles, mental calculations and written calculations), in a group of healthy older people (aged 65–98 years). Sixty older individuals were interviewed with the Cognitive Reserve Index questionnaire (CRIq), and assessed with the Numerical Activities of Daily Living battery (NADL), which included formal tasks on math abilities, an informal test on math, one interview with the participant, and one interview with a relative on the perceived math abilities. We also took into account the years of education, as another proxy for Cognitive Reserve. In the multiple regression analyses on all formal tests, CRIq scores did not significantly predict math performance. Other variables, i.e., years of education and Mini-Mental State Examination score, accounted better for math performance on NADL. Only a subsection of CRIq, CRIq-Working-activity, was found to predict performance on a NADL subtest assessing informal use of math in daily life. These results show that education might better explain abstract math functions in late life than other aspects related to Cognitive Reserve, such as lifestyle or occupational attainment.

## Introduction

Nowadays, Cognitive Reserve is considered as an important variable that can explain much of the variation in cognitive performance in older people (e.g., Fratiglioni et al., 2004; Barulli and Stern, 2013). Cognitive reserve has been defined as the “Individual differences in how people process tasks allow some to cope better than others with brain pathology” (Stern, 2009). Cognitive Reserve derives from a broad combination of experiences a person has throughout life, shaped by education and learning in different contexts, and has a prominent role in determining the early impact of degenerative diseases (Fratiglioni and Wang, 2007; Stern, 2009, 2012). Resilience to the devastating effects of brain degeneration is closely related to the amount of Cognitive Reserve. A high Cognitive Reserve may thus explain, for example, the discrepancy between relatively mild clinical manifestations and a severe underlying cerebral pathology (Barulli and Stern, 2013). Cognitive Reserve is a functional concept, but it is correlated with some measures of Brain Reserve i.e., quantity of brain matter or efficiency of brain networks (Steffener and Stern, 2012).

Many investigations indicate Cognitive Reserve as the most reliable factor for predicting the onset of dementia (Fratiglioni et al., 2004). Several studies are currently underway evaluating the involvement of Cognitive Reserve in preserving specific cognitive functions, and, vice versa, to evaluate the importance of single cognitive functions in determining the amount of Cognitive Reserve. In the domain of language, for example, abilities like richness of vocabulary, comprehension and, in particular, bilingualism have been considered. Thus, most studies on bilingualism agree that knowing two or more languages significantly increases Cognitive Reserve, brain capacity, and flexibility, and is correlated with a later dementia onset (Bialystok et al., 2007; Craik et al., 2010). Most researchers would agree that the greater a person's language ability, the lower the likelihood of developing early dementia (Snowdon et al., 1996), although some authors disagree with this view (Chertkow et al., 2010; Zahodne et al., 2014). In most studies in the literature Cognitive Reserve has been operationalized simply using years of education as a proxy (Stern, 2002). However several other aspects appear to contribute to overall Cognitive Reserve, such as occupational attainment (see Stern et al., 1995; Staff et al., 2004; Garibotto et al., 2008), and leisure activities (see Wilson et al., 2002; Scarmeas et al., 2003).

A question arises, however, as to whether distinct cognitive functions are homogeneously sensitive to Cognitive Reserve. Numerical abilities, which have been shown to be organized in a highly modular way (Semenza, 2008; Cappelletti, 2016), are particularly interesting in this respect. A wide number of numerical abilities are needed in everyday life, and even a partial loss or slowing down of these abilities may have significant personal and social consequences. Little is know about how numerical abilities change in aging. A number of recent investigations lead to divergent conclusions, in part depending on which numerical functions were measured. In the elderly, even in healthy ones, a certain degree of impairment of arithmetical functions can be found (Duverne and Lemaire, 2004; El Yagoubi et al., 2005; Lemaire and Arnaud, 2008; Benavides-Varela et al., 2015), which is often clinically hard to distinguish from that produced by neurological deterioration (Zamarian et al., 2007a,b). However, not all studies show an age-related decline. For example, Cappelletti et al. (2014) showed that healthy old people are impaired in numerosity discrimination, while remarkably accurate in performing arithmetical tasks. Moreover, the only deficit found, poor numerosity discrimination, was likely to reflect an older persons impoverished inhibitory processes rather than an authentic numerical impairment. Norris et al. (2015) even showed a positive impact of age in both basic symbolic numerical processing and mathematical abilities.

Rather than investigate simply whether some math abilities increase or decrease with age, the aim of the present study is to investigate to what extent math abilities in aging are correlated with Cognitive Reserve, taking into account demographic variables (i.e., age, sex) and global cognitive functioning (as measured by MMSE). Firstly, we were interested to examine the difference between association of Cognitive Reserve and numerical abilities compared with formal education and numerical abilities, which is acquired in a formal way over a limited period of life. Secondly, we wanted if it was possible to disentangle an effect of other aspects of Cognitive Reserve on math performance from an effect of global cognitive functioning. It is expected that Cognitive Reserve will correlate to some extent with performance on global functioning tests as the MMSE.

In this study the relation of Cognitive Reserve to numerical abilities is investigated distinguishing between the formal use of abstract mathematical abilities and their use in everyday life. To achieve these aims we used two relatively new instruments: the Numerical Activities of Daily Living (NADL, Semenza et al., 2014) and the Cognitive Reserve Index questionnaire (CRIq, Nucci et al., 2012). This latter has been devised to comprehensively capture all possible sources of reserve quickly and efficiently (including education, occupational attainment, leisure time activities, social and physical activities, and intelligence). Thus, the CRIq quantifies and transforms all of the activities that are known to be strictly associated with Cognitive Reserve into scores. In addition to CRIq we also included education as a further measure, to assess the part of cognitive reserve that is associated with formal schooling. Years of education is indeed the most commonly used proxy for cognitive reserve in most studies (e.g., Farfel et al., 2013; López et al., 2014).

## Methods

### Participants

A sample of 60 older individuals (aged 65–98 years) took part voluntarily in the study. All participants were autonomous in their activity of daily living, had no relevant neurological, or psychiatric disease that could affect cognitive performance, and had a Mini-Mental State Examination (MMSE, Folstein et al., 1975) score above 24. The mean age of participants was 73.25 years (SD = 7.14) and their mean education was 10.97 years (SD = 4.56). Among the participants 34 were female and 26 male. They varied widely in terms of occupation. Detailed descriptive statistics are reported in Table 1. Details on Job are reported in the Supplementary Data Sheet 1.

**Table 1:** Descriptive Statistics.

**Variable**	**Mean**	***SD***	**Median**	**Min**	**Max**	**Skeweness**	**Kurtosis**	**1st Quartile**	**3rd Quartile**
Age	73.25	7.14	72.5	65	98	0.93	0.84	67.75	77.25
Years of education	10.97	4.56	12	4	24	0.58	0.14	8	13
CRIq-education	108.23	15.51	108	83	149	0.58	−0.16	96	116
CRIq-workingactivity	107.53	20.92	103	71	170	0.53	−0.03	93.5	123.25
CRIq-leisuretime	132.07	20.77	128	78	170	0.02	−0.73	118.5	150.25
CRIq-TOTAL	121.22	18.72	119.5	81	164	0.35	−0.39	107.75	132
MMSE	28.76	1.41	29	24.5	30	−1.28	1.03	28	30
NADL-patient interview	8.97	0.88	9	6	10	−0.66	0.47	8	10
NADL-relative interview	9.12	0.92	9	6	10	−0.99	0.79	9	10
NADL-informal test on numerical competence	21.77	1.67	22	16	23	−1.65	2.12	21.75	23
NADL-total number comprehension	17.65	1.04	18	16	19	−0.08	−1.23	17	19
NADL-total reading and writing arabic numerals	9.58	1.12	10	5	10	−3.37	10.83	10	10
NADL-total mental calculation	17.2	1.67	18	11	18	−2.28	4.74	17	18
NADL-total rules and principles	11.62	2.64	12	3	15	−0.94	0.87	10	13.25
NADL-total written operations	14.4	3.6	15	0	17	−2.6	7.36	13	17

*Means, standard deviations, median, minimum, maximum, skewness, kurtosis, first quartile, and third quartile for all variables included in the study (number of participants = 60)*.

### Materials and experimental variables

All the participants were administered the following tests: the Italian version of MMSE (Folstein et al., 1975), the Cognitive Reserve Index questionnaire (CRIq, Nucci et al., 2012), and the Numerical Activities of Daily Living (NADL, Semenza et al., 2014).

CRIq is a semi-structured interview (free download at http://www.cognitivereserveindex.org) which gathers and quantifies all the cognitive reserve related experiences the individual has acquired throughout his/her life. Other than demographic data, the CRIq includes information on three sub-sections: education (CRIq-Education), type of occupation (CRIq-WorkingActivity), and all free-time activities (CRIq-LeisureTime). CRIq-Education refers to a person's school years and any other educational activity (e.g., learning to play a musical instrument or speak a foreign language). CRIq-WorkingActivity classifies five occupational categories with different degrees of responsibility and demands. The number of years spent in each occupation is considered. CRIq-LeisureTime collects information regarding free time activities (for example, sporting activities, reading books, watching television, attending concerts, and so on). The information collected in these three sections of the questionnaire does not seem to be redundant (low correlation among these three areas has been reported, see Nucci et al., 2012), thus the three proxies can be considered independent of one another. The CRIq total score is calculated combining the three sub-scores of each section and is adjusted for age (via a regression based method, see Nucci et al., 2012) to allow comparisons between groups of different ages. The questionnaire can be administered in about 10–15 min and, since it is not a performance test, a patient's caregiver can provide the information in cases where the patient has memory loss or poor comprehension.

Years of Education, the other proxy used for cognitive reserve, was measured as the number of years of formal schooling successfully attended.

NADL measures the impact of specific numerical abilities on participants' everyday life, and has been used to assess the magnitude of numerical deficits in other clinical populations (e.g., Benavides-Varela et al., 2015). A full description, the testing material, the scoring methods and cut-offs for each subtest can be found in Semenza et al. (2014). Briefly, NADL encompasses:

– A short Interview with the participant, which assesses awareness of their possible numerical deficits in everyday life. This interview consists of 10 simple questions enquiring about how well the participant uses numbers in everyday life. (e.g., “Do you shop by yourself?”; “Do you make your own telephone calls unaided, do you dial the number yourself?”). The same interview was also given to a relative of the participant (Relative Interview) for comparison with the participant's own assessment (maximum score: 10).
– An Informal test of numerical competence, which measures the participant's numerical proficiency in everyday tasks. It encompasses questions in the domains of Time (e.g., current date), Measure (e.g., amount of pasta or rice in an average portion), Transportation (e.g., distance between home and hospital), Communication (e.g., own telephone number), General Knowledge (e.g., number of days in a week), and Money (e.g., estimating the price of a new car) (maximum score: 23).
– A Formal Test of Numerical Abilities, which assesses the patient's numerical abilities by using brief sub-tests graded for difficulty. It includes the following domains: Number comprehension (maximum score: 15), Transcoding (reading and writing Arabic numerals; maximum score: 10), Mental calculation (maximum score: 18), Knowledge of rules and properties of calculation (maximum score: 17), and Written operations [e.g., Addition (e.g., 463 + 659), Subtraction (e.g., 548–231), and Multiplication (e.g., 429 × 53)] (maximum score: 17).

While we collected (and analyzed) data on all the three components of NADL for the sake of completeness, it is important to point out that only the Formal Test of Numerical Abilities is directly relevant for the issue addressed in this research, i.e., whether Cognitive Reserve is protective of basic math abilities. The informal test of numerical competence may instead, by its very nature, be related to Cognitive Reserve.

To investigate the effect of job experience we coded a factor with two levels, labeled Job Type: job involving the use of math/job not involving the use of math. Each participant classification was agreed by all the authors. In eight cases, agreement was not reached and those participants were coded as “missing data” for the Job Type (this means that they are excluded from the analysis when the variable Job Type is taken into account). For example, being a cashier in a drugstore was classified by all of the authors as a job which involved math and numbers.

### Procedure

The tests were administered in a fixed order, starting with CRIq, followed by MMSE and then by NADL. Before test administration, written and oral informed consent was obtained from each participant. The NADL relative interview was administered to a close relative of each participant. The study was approved by the Local Ethical Committee of the School of Psychology (University of Padova) and conducted in accordance with the principles of the Declaration of Helsinki.

### Statistical analyses

The relationships between the variables included in the study were examined using a series of pairwise Pearson's correlations. Given the exploratory nature of this analysis, no multiple comparison correction was applied. Importantly, the aim of this analysis was to provide a generic picture of the pairwise relationships between scores, rather than making inferences about those relations.

To obtain more robust conclusions the relationship between the CRIq subsets and performance on specific items of the NADL was then investigated with a series of multiple regressions (R Development Core Team, 2016). Eight separate models were fitted, one for each NADL section as dependent variable: Participant Interview, Relative Interview, Informal Test of Numerical Competence, Total Number Comprehension, Total Reading and Writing Arabic Numerals, Total Mental Calculation, Total Rules and Principles, Total Written Operations.

The predictors used in the each of the regressions were the following continuous variables: CRIq-Education, CRIq-WorkingActivity, CRIq-LeisureTime, age, MMSE raw score, Years of education. Sex (male/female) was included as a factor. Finally, Job Type was included as a factor (see Materials section).

In the regression analysis we did not include the CRIq-Total as it is a score derived mathematically from CRIq-Working Activity, CRIq-Education, and CRIq-LeisureTime, and highly correlated with these scores. In summary, each regression model had a NADL score as dependent variable, and CRIq scores, Age, MMSE, Years of education, and Job type, as predictors.

Before running the regression analysis, the potential presence of harmful collinearity across continuous predictors was checked. Collinearity (i.e., excessive correlations) between predictors may have negative effects on the results of regressions. If two predictors are too much correlated one with the other it is difficult to tease apart the two effects and, as a consequence, the estimate of the regression coefficients of these effects is unstable. To investigate the collinearity across predictors, we checked the Variance Inflation Factor (VIF) associated to each predictor. As a rule-of-thumb, a VIF > 10 on a predictor indicates a harmful collinearity. All predictors had a VIF below 2, with the exception of Education and CRIq-Education, which showed a VIF of 11.63, and 11.83 respectively. This is not surprising since CRIq-Education is obtained as a standardized sum of Years of education, with the exception that an additional 0.5 is added to the score for every 6 months of training courses (see Nucci et al., 2012 for details). Thus, Years of education and CRIq-Education tend to be very highly correlated, as is the case for our sample, in which the correlations between these variables showed a Pearson coefficient of 0.93 (See Table 2). The detrimental effect of this collinearity is partly avoided by the variable selection method we employed, that is a stepwise forward method. In this method, starting from an empty model, predictors are added one at a time, including the predictors that lead to the best fitting model. As CRIq-Education and Years of education were correlated, we expected that only the one that better accounted for the data would be first included in the regression model and then kept in the final model.

**Table 2:** Correlation matrix.

	**Age**	**Years of education**	**CRIq-education**	**CRIq-Working Activity**	**CRIq-Total**	**MMSE**	**NADL–patient interview**	**NADL–relative interview**	**NADL–informal test on numerical competence**	**NADL-total number comprehension**	**NADL–total reading and writing arabic numerals**	**NADL–total mental calculation**	**NADL–total rules and principles**	**NADL–total written operations**
Age	1	−0.19	0.04	0.02	−0.06	−0.38^*^	0.01	−0.09	−0.29^*^	−0.18	−0.58^*^	0	−0.44^*^	−0.27^*^
Years of education	−0.19	1	0.93^*^	0.3^*^	0.34^*^	0.48^*^	0.32^*^	0.36^*^	0.29^*^	0.32^*^	0.16	0.26^*^	0.52^*^	0.33^*^
CRIq-education	0.04	0.93^*^	1	0.36^*^	0.35^*^	0.39^*^	0.22	0.31^*^	0.25	0.25^*^	0.01	0.26^*^	0.39^*^	0.26^*^
CRIq-workingactivity	0.02	0.3^*^	0.36^*^	1	0.28^*^	0.18	0.03	0.14	0.29^*^	0.17	0.08	0.08	0.26^*^	0.06
CRIq-Total	−0.06	0.34^*^	0.35^*^	0.28^*^	1	0.21	0.21	0.26^*^	0.3^*^	0.3^*^	0.06	0.18	0.31^*^	0.21
MMSE	−0.38^*^	0.48^*^	0.39^*^	0.18	0.21	1	0.1	0.21	0.44^*^	0.42^*^	0.25	0.38^*^	0.53^*^	0.58^*^
NADL–patient interview	0.01	0.32^*^	0.22	0.03	0.21	0.1	1	0.84^*^	−0.02	0.19	−0.01	0.05	0.16	0.13
NADL–relative interview	−0.09	0.36^*^	0.31^*^	0.14	0.26^*^	0.21	0.84^*^	1	0.15	0.22	0.03	0.15	0.28^*^	0.2
NADL–informal test on numerical competence	−0.29^*^	0.29^*^	0.25	0.29^*^	0.3^*^	0.44^*^	−0.02	0.15	1	0.4^*^	0.2	0.29^*^	0.59^*^	0.33^*^
NADL–total number comprehension	−0.18	0.32^*^	0.25^*^	0.17	0.3^*^	0.42^*^	0.19	0.22	0.4^*^	1	0.21	0.31^*^	0.39^*^	0.38^*^
NADL–total reading and writing arabic numerals	−0.58^*^	0.16	0.01	0.08	0.06	0.25	−0.01	0.03	0.2	0.21	1	0.09	0.38^*^	0.07
NADL–total mental calculation	0	0.26^*^	0.26^*^	0.08	0.18	0.38^*^	0.05	0.15	0.29^*^	0.31^*^	0.09	1	0.48^*^	0.65^*^
NADL–total rules and principles	−0.44^*^	0.52^*^	0.39^*^	0.26^*^	0.31^*^	0.53^*^	0.16	0.28^*^	0.59^*^	0.39^*^	0.38^*^	0.48^*^	1	0.6^*^
NADL–total written operations	−0.27^*^	0.33^*^	0.26^*^	0.06	0.21	0.58^*^	0.13	0.2	0.33^*^	0.38^*^	0.07	0.65^*^	0.6^*^	1

The Table reports all the pairwise correlations (Pearson's r coefficients) between the variables included in the study. Asterisks (

* *) denote correlations with p < 0.05*.

Specifically, for the forward selection of variables we used the R *step* function, which starting from a null model including only the intercept adds one predictor at a time, starting from the one whose inclusion leads to the model with the best Akaike Information Criterion (AIC). Since the Job Type variable included some missing data (see above) it was introduced at the end of the procedure, after checking the effects of all the other variables. After this variable selection based on AIC, predictors included whose *p*-values were above 0.05 were excluded one at a time, starting from the one with the highest *p*-value. Following this procedure, each final regression model included only the significant variables that best accounted for the dependent variable. To ensure reliability of results, after a final model was defined, partial residuals of all effects were inspected to assess if adding non-linear terms could improve the fit. Moreover, we inspected the presence of outliers that could have influenced the regression estimates. In such cases we re-fitted the models after excluding the outliers. The percentage of explained variance of each model was calculated as adjusted *R*^2^.

## Results

Descriptive statistics of CRIq, MMSE, and NADL scores are reported in Table 1. Performance in scores NADL battery was variable across participants and only relatively few participants performed at ceiling. In almost all NADL tasks the distribution of the scores showed left skeweness (as expected in the scores on neuropsychological test in a healthy sample). Scores in CRIq sections spanned across different values and showed right skewed distribution for CRIq-WorkingActivity and CRIq-Education, whereas CRIq LeisureTime showed an almost symmetrical distribution.

All pairwise correlations between the variables included in the study are reported in Table 2. Importantly for the aims of the present study, CRIq variables showed several significant (moderate or weak) correlations with NADL performance. In particular, CRIq-Education correlated with the Relative Interview [*r*_(58)_ = 0.31, *p* = 0.01], Total Number Comprehension [*r*_(58)_ = 0.25, *p* = 0.01], Total Mental Calculation [*r*_(58)_ = 0.26, *p* = 0.4], Total Rules and Principles [*r*_(58)_ = 0.39, *p* = 0.002], Total Written Operations, [*r*_(58)_ = 0.26, *p* = 0.04]. CRIq-WorkingActivity correlated with the Informal Test on Numerical Competence [*r*_(58)_ = 0.29, *p* = 0.03], and the Rules and Principles [*r*_(58)_ = 0.26, *p* = 0.04]. CRIq-LeisureTime correlated with Relative Interview [*r*_(58)_ = 0.26, *p* = 0.04], the Informal Test on Numerical Competence [*r*_(58)_ = 0.30, *p* = 0.02], Total Number Comprehension [*r*_(58)_ = 0.30, *p* = 0.02], and Rules and Principles [*r*_(58)_ = 0.31, *p* = 0.02].

To better understand the relationship between CRIq and NADL, we performed a series of multiple regressions with NADL scores as dependent variables, including as predictors the CRIq indices and other variables, i.e., Age, Years of education, and MMSE (see Methods for details). In all regression models at least one significant predictor was found. In no case it was necessary to add non-linear terms to improve the fit. During the last section of modeling procedure, some outliers were detected in the model on Informal Test on Numerical Competence (four outliers), Total Mental Calculation (three outliers), and Total Written Operations (one outlier). In such cases the removal of outliers did not affect the significance of the effects and caused only small differences in regression estimates. For the models in which some outliers were identified, only the results after outlier removal are reported.

### Patient interview and relative interview

In the analysis of the model with the Patient Interview the final model included only a positive effect of education (*Intercept* = 8.3, SE = 0.29, *t* = 29, *p* < 0.001; *Years of Education* = 0.06, SE = 0.02, *t* = 2.53, *p* = 0.01, *R*^2^ = 0.08), indicating that as Years of education increases the predicted score on Patient Interview is higher. Similar results were found in the model with the Relative Interview, in which only a positive effect of education was found (*Intercept* = 8.3, SE = 0.29, *t* = 28, *p* < 0.001; *Years of education* = 0.07, SE = 0.02, *t* = 2.97, *p* = 0.004, *R*^2^ = 0.11).

### Informal test of numerical competence

In the model with Informal Test of Numerical Competence, two significant effects were found: a positive effect of MMSE and a positive effect of CRIq-Working Activity (*Intercept* = 8.6, SE = 2.5, *t* = 3.4, *p* = 0.001; *MMSE* = 0.42, SE = 0.09, *t* = 4.67, *p* < 0.001; *CRIq-WorkingActivity* = 0.01, SE = 0.006, *t* = 2.16, *p* = 0.03; *R*^2^ = 0.35).

### Formal tests of numerical competence

**Figure 1:**
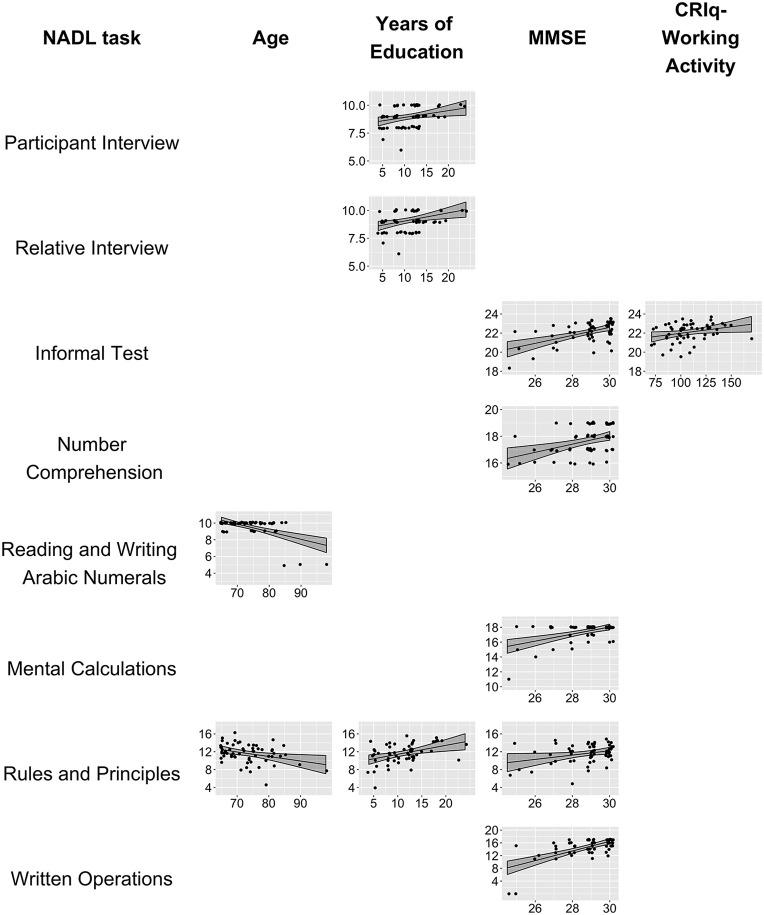
Partial effects of regression models. The figure shows the partial effects of the significant terms of the regression models with NADL scores as dependent variables. The figure displays: the NADL-F task names (first column), the effect of age (second column), the effect of Years of education (third column), the effect of MMSE (fourth column), and the effect of CRIq-WorkingActivity (fifth column). Effects not displayed were not significant in the regression analyses. The black line in each plot represents the predicted score at the task. The gray bands around the lines represent point-wise confidence bands around the prediction. The black dots indicate the observed data for each participant (each dot represent a participant). A small jitter was applied to the observed values for a better visualization.

In the model with the Total Number Comprehension Score, only the MMSE was listed as a significant predictor: as the MMSE score increases the predicted score on Total Number Comprehension increases (*Intercept* = 8.8, SE = 2.5, *t* = 3.5, *p* < 0.001; *MMSE* = 0.32, SE = 0.08, *t* = 3.49, *p* < 0.001, *R*^2^ = 0.16). In the analysis on Total Reading and Writing Arabic Numerals a negative effect of age was found (*Intercept* = 16, SE = 1.2, *t* = 13.09, *p* < 0.001; *Age* = −0.09, SE = 0.02, *t* = −5.40, *p* < 0.001, *R*^2^ = 0.32), with a significantly worse performance as the Age increases. For the Total Mental Calculation, again a positive effect of MMSE was found (*Intercept* = 3.79, SE = 2.96, *t* = 1.28, *p* = 0.21; *MMSE* = 0.48, SE = 0.10, *t* = 4.62, *p* < 0.001, *R*^2^ = 0.27). The final model on Rules and Principles total scores included three significant effects: a positive effect of MMSE, a positive effect of education, and a negative effect of age (*Intercept* = 2.9, SE = 7.82, *t* = 0.37, *p* = 0.71; *MMSE* = 0.48, SE = 0.23, *t* = 2.12, *p* = 0.03; *Years of Education* = 0.20, SE = 0.07, *t* = 3.04, *p* = 0.004; *Age* = −0.10, SE = 0.04, *t* = −2.52, *p* = 0.015, *R*^2^ = 0.41). Finally, the regression model on Total Written Operation evidenced only a significant effect of MMSE (*Intercept* = −28.93, SE = 6.74, *t* = 4.29, *p* < 0.001; *MMSE* = 1.51, SE = 0.23, *t* = 6.46, *p* < 0.001, *R*^2^ = 0.41). Partial effects of regression models are shown in Figure 1.

The results of the regression analysis showed that each NADL scores is affected by different variables. Three tests, i.e., Total Number Comprehension, Total Mental Calculation, and Total Written Operations, were influenced only by MMSE. In all these tests higher scores at MMSE were associated with better performance in the NADL total scores.

The two NADL Interviews (with the Patient and with the Relative) were influenced only by Years of Education, with better reported numerical abilities as education increases.

The Reading and Writing Arabic Numerals was affected only by Age, with lower scores as Age increases. Finally, only two scores showed the effect of more than one variable. The model on Rules and Principles showed significant positive effects of MMSE and Years of Education, and a negative effect of Age. The Informal Test on Numerical Competence showed a significant effect of CRIq-WorkingActivity, together with a positive effect of MMSE.

Several variables were excluded in the final models identified because, according to the variable selection employed, their effects on the dependent variable were negligible. This indicates that Sex and Job Type did not have any relevant effect in predicting NADL scores. Moreover, it indicates that, apart from the effect of CRIq-WorkingActivity on the Informal Test on Numerical Competence, the CRIq variables did not have any important influence on the performance of older people on NADL subtests.

## Discussion

The object of the current study was to find what role Cognitive Reserve has in maintaining good mathematical abilities in aging while taking into account the effects of variables such as education and general cognitive status. To measure Cognitive Reserve we used the Years of education and the CRIq, a questionnaire investigating characteristics of the individual that are known to be associated with cognitive reserve (Nucci et al., 2012). To measure mathematical abilities we used NADL (Semenza et al., 2014), a test that assesses both formal mathematical abilities and informal aspects such as the use of math in daily life. On the whole, both Years of education and CRIq indices, when taken independently, correlated with several NADL scores. However, when entered into a model taking into account other variables, the CRIq indices showed little or no relationship to the NADL scores, with the exception of the Informal Test section. Thus, the main result of this study is that the amount of Cognitive Reserve, as estimated by the CRIq, is not associated with the efficiency of formal mathematical abilities in healthy aging. Rather, our findings suggest that other variables, such as Years of Education, or global cognitive functioning, may better account for performance on the formal section of NADL.

These results suggest that at least some conceptualizations of Cognitive Reserve need better characterization. As the present findings show, distinct cognitive functions are not homogeneously sensitive to Cognitive Reserve (see for example Clare et al., 2017 for a study with positive correlations of Cognitive Reserve in a generic cognitive battery, or Puccioni and Vallesi, 2012, for correlation of Cognitive Reserve with conflict resolution abilities). Thus, the problem arises of what makes a given cognitive function more or less sensitive, if at all, to the amount of Cognitive Reserve, if considering the sum of several aspects (such as education, occupational attainments, and life styles). This study reveals that abstract math functions, even when considered separately according to their modular organization, do not seem to be strongly associated with several aspects of Cognitive Reserve, with the exception of Years of education.

This is interesting insofar as it suggests the teasing apart of the relative role of education in Cognitive Reserve. Education refers to a particular type of experience acquired very early in life and consists of intensive, formal, learning (indeed, for the majority of our Italian participants it meant attending 5 years of Elementary School, from the age of 6 to the age of 10). Cognitive Reserve as measured by CRIq, comes instead from the vast range of experiences and learning that a person gathers throughout life. Experience constituting Cognitive Reserve is mostly acquired in a different, less systematic way with respect to the experience derived from education. On one hand, Cognitive Reserve seems to protect the individual from a general cognitive decline (e.g., Stern, 2009), by concurring to find flexible solutions to everyday problems, as it seems to be demonstrated here by the effect on NADL Informal Test. On the other hand, Cognitive Reserve appears to be less effective when specific and systematically learnt knowledge comes into play. It is worth noting that Years of education does not necessarily predict cognitive performance changes with aging. For example, MacPherson et al. (2016) found that education does not predict changes in fluid intelligence as well as in verbal short term memory, perceptual abilities, and processing speed.

There are also some other considerations that can be made from the results of the present study. First, results showed that only specific aspects of Cognitive Reserve could be associated with cognitive performance, rather than Cognitive Reserve *per se*. This is the case of the widespread effect of education that we found in the results, as compared with the scarce effects of other variables (such as job type and job experience). Although we acknowledge the clinical and practical importance of using general proxies to measure Cognitive Reserve (as the CRIqTotal score) our results show that for research purposes it may be important to consider separately the different variables that may contribute to Cognitive Reserve.

Moreover, results from the present study stress the importance of a distinction between a broader conceptualization of Cognitive Reserve (measured by several proxies tapping different aspects, as CRIq) and education. Years of education is the most common proxy used to measure cognitive reserve (see for example Farfel et al., 2013; López et al., 2014; Sumowski et al., 2014). The results of the current study highlight that the two concepts can, and should, be separated: the result that education is associated with better cognitive functioning does not necessarily imply that all Cognitive Reserve is. At least for some math abilities, only education and not other aspect of Cognitive Reserve (such as the leisure time activity or work experience) are associated with better performance. In other words, concluding that Cognitive Reserve has some effect on cognitive performance just because education did can be an overgeneralization of a more specific effect.

There are some important caveats for the present study that need to be highlighted. First, it is important to underline that we may only speculate on a causal relationship between education and math abilities in old age, given the correlational nature of the collected data. Moreover, we did not find any specific effect of job type on mathematical abilities in aging. Importantly, this could be related to the way we defined the Job Type variable. As assessing the role of job experience was not one of the specific aims of the study, we did not separate people who perform simple math in every day life (such as as cashiers or accountants) from people who work daily with high-level math (such as high-school math teachers, engineers, physicists, etc.). A study focusing on the effects of these different working backgrounds could shed better light on the role of job experience on late life math abilities. Another limitation concerns the tools employed. Although, both the Criq and the NADL were both successfully employed in several studies (e.g., Mondini et al., 2016; Benavides-Varela et al., 2017; Burgio et al., 2017) they are just proxies for Cognitive Reserve and math abilities, respectively. For this reason, further studies employing different tools are necessary to strengthen the present results. An important note concerns the design employed: as the present study is cross-sectional it cannot fully explore the effect of cognitive reserve on math abilities. Only a longitudinal study (possibly a large scale study) can really confirm that only education (and not Cognitive Reserve in general) can protect against a decline in math abilities. As showed by Cadar et al. (2017), education may be associated with better memory performance when studied cross-sectionally but longitudinally it has limited protective effects on age-related deterioration.

Finally, as the present study does not include data on brain structure and integrity, we can only speculate about the actual compensation and effect that cognitive reserve may have on brain decay. A relatively recent study by Zahodne et al. (2015) showed how brain and cognitive data may be integrated to provide a better proxy for Cognitive Reserve.

Summarizing, in the present study we investigated the association between performance in math abilities (as measured by NADL), Cognitive Reserve (as measured by CRIq and Years of Education) and other variables, general cognitive functioning (measured by MMSE), Age. Our results showed that several aspects known to be associated to Cognitive Reserve were weakly associated with math performance, whereas MMSE and Years of education, were the strongest predictors. These results suggest the importance of early formal schooling and general cognitive status in math performance in older age, rather than the importance of a broader concept of Cognitive Reserve. Assessing how Cognitive Reserve influences the maintenance of differently acquired types of abilities in aging seems indeed a promising field for future investigation.

## Ethics statement

The study was approved by the Local Ethical Committee of the School of Psychology (University of Padua) and conducted in accordance with the principles of the Declaration of Helsinki. Protocol n. 2068, ID:FDDFA2B003E72157CA89E79A053722C7.

## Author contributions

SM, CS, GA, and FM designed the study. AB collected the data. FM supervised the data collection. GA performed the analysis and prepared the tables and figures. SM, CS, GA, and KP interpreted the results and wrote the manuscript.

### Conflict of interest statement

The authors declare that the research was conducted in the absence of any commercial or financial relationships that could be construed as a potential conflict of interest.

## Supplementary material

Supplementary Data Sheet 1 The supplementary data contains a list of the jobs of the participants included in the study.
